# Adherence to dietary guidelines associated with lower medical service utilization in preschoolers: a longitudinal study

**DOI:** 10.1038/s41387-024-00270-w

**Published:** 2024-03-22

**Authors:** Yi-Chieh Chen, Yuan-Ting C. Lo, Hsin-Yun Wu, Yi-Chen Huang

**Affiliations:** 1 Department of Nutrition, China Medical University, No. 100, Sec. 1, Jingmao Rd., Beitun District, Taichung, 406040 Taiwan; 2 Department of Nutrition, Chang Bing Show Chwan Memorial Hospital, No. 6, Lugong Rd., Lukang Township, Changhua County, 50544 Taiwan; 3 Department of Public Health, National Defense Medical Center, No. 161, Sec. 6, Minquan E. Rd., Niehu District, Taipei, 11490 Taiwan

**Keywords:** Paediatrics, Epidemiology

## Abstract

**Objective:**

We aimed to evaluate the association between dietary guideline adherence and overall, outpatient, and emergency medical service utilization in Taiwanese preschoolers.

**Methods:**

We selected 614 preschoolers (2–6 years) who had one day of 24-h dietary recall data from the 2013–2016 Nutrition and Health Survey in Taiwan. The Taiwanese Children Healthy Eating Index (TCHEI) was developed on the basis of Taiwanese Food-Based Dietary Guidelines; it assesses dietary adequacy and eating behavior. Data on the participants’ outpatient and emergency medical service utilization were obtained for 2013–2018 from the National Health Insurance Research Database. A multivariable generalized linear model was used to evaluate the association between the TCHEI and medical service utilization for all disease and respiratory diseases.

**Results:**

After adjustment for confounding factors, children aged 2–3 years in the Tertile (T) 2 and T3 groups of the TCHEI exhibited 25% (95% CI 0.69–0.83) and 16% (95% CI 0.77–0.92) lower overall medical visits, respectively. The same pattern was noted in the outpatient and emergency visits for all diseases and respiratory diseases. The children aged 4–6 years in the T2 group exhibited 15% (95% CI 0.80–0.91) and 11% (95% CI 0.82–0.97) lower overall visits and visits for respiratory diseases, respectively. Moreover, preschoolers in the T2 group exhibited lower overall medical expenditures than did those in the T1 group.

**Conclusions:**

TCHEI score was positively correlated with better nutritional status. Optimal dietary intake associated with lower medical service utilization among Taiwan preschoolers.

## Background

Early childhood is one of the three rapid growth periods in life that is critical for the development of physiological, cognitive, and immune functions [1, 2]. Moreover, the transition from liquid food to solid food represents a critical phase in the establishment of personal eating behaviors during this period. A healthy diet provides adequate nutrients to fulfill human growth needs and prevent chronic diseases in later life [3]. Undernutrition is detrimental to the development of the neurocognitive system of preschoolers and thus affects their social interactions [2]. However, excessive calorie intake and an unhealthy diet increase the risk of obesity and related noncommunicable chronic diseases, thereby increasing the risk of illness in adulthood [4].

A healthy diet consists of consuming a variety of food, moderate food intake and optimal dietary behavior are essential for obtaining adequate nutrition [5]. Studies have reported that children’s dietary quality declines from the age of 6 months to 4 years [6]. This may be because, by this age, children begin to consume processed food or beverages of their own choice. Therefore, various countries have encouraged the development of food-based dietary guidelines for children and the education of the public to adopt healthy diets in their daily lives; such practices can promote health and prevent chronic diseases [7]. Understanding the overall diet quality rather than evaluating the contribution of a single nutrient is critical for health promotion in early childhood. A healthy diet strengthens the immune system; for example, the phytochemicals in fruits and vegetables can reduce oxidative stress, thereby reducing the chances of inflammation [8, 9]. In contrast, adopting a Western diet increases the risks of asthma and respiratory infections in preschoolers [10]. Despite this, few studies have focused on the cost-effectiveness of promoting adherence to dietary guidelines among preschoolers.

Medical service utilization can be used as a surrogate means of examining a person’s overall health and evaluating the cost-effectiveness of health promotion strategies. Kirk et al. reported that a higher healthy eating score resulted in lower medical service utilization among Canadian elementary school children, but the findings were not statistically significant due to the low medical visit rate in this population [11]. Taiwan’s National Health Insurance (NHI) program is compulsory for all residents to eliminate health inequity; the program records each person’s medical service utilization and expenditures [12]. In Taiwan, the outpatient visit rate for the age group of 0–14 years is ranked second followed by older adults. Moreover, among diseases, the outpatient visit rate for respiratory diseases is highest for all age groups [13]. However, whether adherence to food-based dietary guidelines and a healthy diet during the transition period of weaning to primary school can reduce medical service utilization remains unclear.

We developed a Taiwanese Children Healthy Eating Index (TCHEI) on the basis of the Taiwanese Food-Based Dietary Guidelines (TFBDG) and validated the index by assessing the dietary nutrient intake of preschoolers. Subsequently, we investigated the association between the TCHEI and medical service utilization for all diseases, especially respiratory diseases, in Taiwanese preschoolers.

## Methods

### Study participants

Participants’ data were extracted from the Nutrition and Health Survey in Taiwan (NAHSIT) 2013–2016. The survey adopted a multistage, stratified, clustered, probability sampling method to classify Taiwan into 20 strata according to counties and cities. The size of each stratum was proportional to the probability. The details of the sampling design have been published elsewhere [14]. Full-time trainees collected data on baseline demographics, dietary intake, and medical history through face-to-face interviews with the participants’ parents or primary caregivers. A total of 1295 children aged 2–6 years (2–3 years: *n* = 370; 4–6 years: *n* = 925) recruited in the NAHSIT. Of these, 614 participants agreed to link their NAHSIT data to the NHI dataset to enable an assessment of their medical service utilization. No statistically significant difference in demographic variables between the study population and entire dataset (614 children vs. 1295 children; Supplementary Table 1) was observed. All informed consent forms were signed by the participants’ parents. The survey method was approved by the Institutional Review Board of Academia Sinica, Taiwan and protocol of this study was approved by the Central Regional Research Ethics Committee, China Medical University (No. CRREC-108-157).

### NAHSIT dietary data

Dietary intake data were collected using the 24-h dietary recall method. In this method, face-to-face interviews were conducted to collect data on all foods, including drinking water and beverages, consumed by the participant in the past 24 h. Food models, measuring cups, measuring spoons, and other auxiliary tools were used to assist the participants to estimate food intake weight. We classified each food item and calculated the serving size into six food groups reported by the participants [14, 15]. The standardized serving size of six food groups were (1) grains: 1 serving per 15 g of carbohydrate (e.g., 40 g of cooked rice); (2) soybean, fish, eggs, and meat: 1 serving per 7 g of protein (e.g., 30 g of chicken); (3) dairy: 1 serving per 8 g of protein (e.g., 240 mL of milk); (4) vegetables: 1 serving per 25 calories (100 g of cauliflower); (5) fruits: 1 serving per 60 calories (e.g., 130 g of apple); and (6) oils and nuts: 1 serving per 5 g of fat (e.g., 5 g of olive oil).

Furthermore, the weight of individual food items was converted into nutrient intake according to the Taiwan Food Nutrient Composition Database [16] and the United States Department of Agriculture Food Composition Database [17]. We calculated nutrient intake per 1000 kilocalories to reduce the deviation caused by total energy intake.

### TCHEI development

The TCHEI assesses dietary adequacy and dietary behavior; the total score of the index is 100 points. The dietary adequacy was developed on the basis of the recommended intake amount of grains; soy, fish, eggs, and meat; dairy; vegetables; fruits; and oils and nuts by the TFBDG for children aged 2–6 years with low physical activity [15]. Each food group were given a minimum of 0 points to a maximum of 10 points, and points were assigned in proportion to the standard food intake (Table 1). Furthermore, unrefined grains, soy products, and dark vegetables assigned extra points if they consumed over one-third of their respective categories: grains, soy/fish/eggs/meat, and vegetables. Points for each category were assigned on a scale from 0 to 5. The overall maximum score was 75 points.

**Table 1:** Criteria of Taiwanese Children Health Eating Index among preschool children.

Items	Score range	Standard			
		2–3 years		4–6 years	
		Min	Max	Min	Max
Dietary adequacy (serving)					
Total grains	0–10	0	≥1.5	0	Girl ≥2; boy ≥2.5
Unrefined grains	0–5	0	≥1/3 from unrefined grain	0	≥1/3 from unrefined grain
Soy/fish/eggs/meat	0–10	0	≥2	0	≥3
Soy products	0–5	0	≥1/3 from soy products	0	≥1/3 from soy products
Dairy	0–10	0	≥2	0	≥2
Vegetables	0–10	0	≥2	0	≥3
Dark vegetables	0–5	0	≥1/3 from dark vegetable	0	≥1/3 from dark vegetable
Fruits	0–10	0	≥2	0	≥2
Oils and nuts	0–10	0	≥4	0	≥4
Dietary behavior (times/day)					
Eating breakfast	0–5	0	1	0	1
Frequency of SSB	0–5	≥1	0	≥1	0
Frequency of fried food	0–5	≥1	0	≥1	0
Frequency of snack/ biscuits	0–5	≥1	0	≥1	0
Frequency of seasoning	0–5	≥1	0	≥1	0

*SSB* sugar-sweetened beverages. Unrefined grain: brown rice, germ rice, whole wheat products, potatoes and taro, etc.

An optimal dietary behavior was defined on the basis of the TFBDG recommendation and the Taiwanese version of the Youth Healthy Eating Index [18]. Dietary behavior included the frequency of (1) eating breakfast, (2) sugar-sweetened beverage (SSB) consumption, (3) fried food intake, (4) biscuit or snack intake, and (5) seasoning use. Each item of dietary behavior was scored 0–5 points, for a total of 25 points. The detailed scoring criteria of TCHEI are provided in Table 1.

### Medical service utilization

The National Health Insurance Research Database (NHIRD) was provided by the Health and Welfare Data Science Center, Taiwan. Medical service utilization that is recorded by the medical staff for each visit of insurant. We linked the NAHSIT 2013–2016 data to the 2013–2018 NHIRD data by using scramble IDs. We considered the annual medical service and medical expenditure as an outpatient visit or an emergency visit for consulting a physician. If there were one or more visits on the same day, the number of visits was considered one. Respiratory medical service utilization was defined using *the International Classification of Diseases (ICD), Ninth [Tenth] Revision* (*ICD-9*: 460–466, 470–478, 480–487, 490, and 493) and *ICD-10* (J00–J06, J30–J39, J10–J18, J20–J21, J40, and J45) diagnosis codes. The time of follow-up was calculated from the date of the interview to December 31, 2018. The total medical expenditure was summed of outpatient and emergency expenditure for all medical treatment and medication cost in each visit. The respiratory medical expenditure was summed of the medical treatment and medication cost for each respiratory outpatient and emergency medical service. Annual medical expenditure was calculated by total medical expenditure dividing the follow-up years for each participant. The medical expenditures were adjusted an annual discount rate of 3% and an annual Taiwanese Consumer Price Index from 2013 to 2018 [19].

### Covariables

We calculated sex-specific and age-specific height-for-weight *z* scores (HAZs) and weight-for-age z scores to represent the participants’ nutrition status. We divided the parents’ education levels into junior high school or below, high school, and college or above. The household financial status was evaluated according to parents’ income and expenditure status were divided into enough (had extra money), just enough (no difficulty), some difficulties, and very difficult.

### Statistical analysis

All data analysis was performed using SAS 9.4 (SAS Institute Inc., Cary, North Carolina, USA). We divided the TCHEI scores into three groups by using the tertile (T) distribution. The cutoff points of the TCHEI scores for the participants aged 2–3 years and 4–6 years were 46.3/46.0 and 55.1/54.9, respectively. Categorical variables are presented as *n* (%), and continuous variables as mean ± standard deviation (SD). Except for the percentage of dietary fat intake, all nutrient intake data were non-normally distributed according to the Kolmogorov–Smirnov test. Therefore, analysis of variance and the Kruskal–Wallis test were used to evaluate the distribution difference between fat intake (%) and the intake of remaining nutrients in the TCHEI groups, respectively. The multivariable generalized linear model with a log link and Poisson distribution was used to evaluate the association between the tertile groups of TCHEI scores and medical service utilization for all diseases and respiratory diseases; a log link and gamma distribution was used to evaluate the association between the tertile groups of TCHEI scores and the corresponding medical expenditure. The exponential (exp) β coefficients are presented as a percentage of decrease or increase in medical service utilization compared with the reference group. For considering the differences in development, nutrition, and healthcare service demands, the age-stratification (2–3, 4–6 years old) was used to clarify the effect of dietary quality and medical service utilization. The model was adjusted for confounding factors, including age, sex, region (north, middle, south, east, or island), mother’s education level (junior high school or below, high school, or college or above), family financial status (enough, just enough, some difficulties, or very difficult), and total energy intake by literature review. A two-sided hypothesis test was conducted and *P* < 0.05 was considered statistically significant.

All data analyses of this study are presented unweighted due to the unique data distribution characteristics of medical service and medical expenditure, which does not impact the representativeness of our findings for the study population.

## Results

Supplementary Table 2 provides the overall diet quality score for each component. The mean ± SD TCHEI score was 50.6 ± 10.6. Compared with the participants in the lowest tertile (T1) group, those in the highest tertile (T3) group exhibited a significantly higher score for each component (all *P* < 0.05), except for the frequency of SSB and snack or biscuit consumption.

Table 2 lists the distribution of the participants’ baseline characteristics. The results revealed that the participants in the T3 group (participants with the highest TCHEI scores) were more likely to exhibit a greater HAZ score (0.07 vs. −0.12), higher mother’s education level (59.1% vs. 51.5%), and a better self-reported household financial status (24.8% vs. 12.9%) than those in the T1 group (participants’ with lowest TCHEI scores). Overall, children who lived in the north of Taiwan had higher parental education levels, enough household financial status, and the highest mean TCHEI.

**Table 2:** Baseline characteristics by tertiles of Taiwanese Children Health Eating Index among preschool children (*n* = 614).

	Taiwanese Children Health Eating Index [*n* (%)]				
	Mean ± SD^a^	Total n (%)	Tertile 1	Tertile 2	Tertile 3
TCHEI score, mean ± SD	50.6 ± 10.6		38.7 ± 5.6	50.7 ± 2.5	61.8 ± 5.4
Total *n*		614 (100)	202	202	210
Age (years)					
2–3	50.4 ± 10.8	168 (27.4)	55 (27.2)	55 (27.2)	58 (27.6)
4–6	50.6 ± 10.5	446 (72.6)	147 (72.8)	147 (72.8)	152 (72.4)
Gender					
Boy	50.5 ± 10.2	327 (53.3)	108 (53.5)	110 (54.5)	109 (51.9)
Girl	50.7 ± 11.0	287 (46.7)	94 (46.5)	92 (45.5)	101 (48.0)
Region					
North	51.5 ± 10.6	204 (33.2)	59 (29.2)	64 (31.7)	81 (38.6)
Middle	50.4 ± 10.5	100 (16.3)	39 (19.3)	27 (13.4)	34 (16.2)
South	50.2 ± 10.7	163 (26.6)	50 (24.8)	63 (31.2)	50 (23.8)
East or island	49.7 ± 10.5	147 (23.9)	54 (26.7)	48 (23.8)	45 (21.4)
Height-for-age *z* score			−0.12 ± 1.15	0.07 ± 0.99	0.07 ± 0.86
Weight-for-age *z* score			−0.15 ± 0.94	0.05 ± 1.01	0.004 ± 0.99
Mother’s education level					
Under junior high school	48.2 ± 11.2	68 (11.2)	26 (13.0)	25 (12.3)	17 (8.17)
Senior high school	50.2 ± 10.8	200 (32.8)	71 (35.5)	61 (30.2)	68 (32.7)
Above college	51.3 ± 10.2	342 (56.1)	103 (51.5)	116 (57.4)	123 (59.1)
Father’s education level					
Under junior high school	48.7 ± 11.8	75 (12.6)	29 (14.9)	23 (11.7)	23 (11.1)
Senior high school	50.2 ± 10.9	202 (33.9)	74 (38.1)	61 (31.1)	67 (32.5)
Above college	51.4 ± 10.1	319 (53.5)	91 (46.9)	112 (57.1)	116 (56.3)
Household financial status					
Enough	52.9 ± 9.80	121 (19.9)	26 (12.9)	44 (22.0)	51 (24.8)
Just enough	50.5 ± 10.7	336 (55.3)	112 (55.4)	110 (55.0)	114 (55.3)
Not enough	48.4 ± 10.3	122 (20.1)	51 (25.2)	40 (20.0)	31 (15.0)
Very difficulty	50.1 ± 12.7	29 (4.8)	13 (6.4)	6 (3.0)	10 (4.85)

Data were presented as *n* (%) for categorical variables and mean ± SD for continuous variables.

^a^Mean ± SD of Taiwanese Children Health Eating Index score for each variable.

Table 3 lists the nutrient intake of the tertile groups. The median intake of protein (3.5 g/kg BW vs. 2.2 g/kg BW), polyunsaturated fatty acids (9.23 g/1000 kcal vs. 8.09 g/1000 kcal), dietary fiber (7.2 g/1000 kcal vs. 4.3 g/1000 kcal), vitamin C (76.4 mg/1000 kcal vs. 30.8 mg/1000 kcal), vitamin B6 (0.9 mg/1000 kcal vs. 0.7 mg/1000 kcal), potassium (1280 mg/1000 kcal vs. 978 mg/1000 kcal), calcium (380 mg/1000 kcal vs. 224 mg/1000 kcal), magnesium (139 mg/1000 kcal vs. 110 mg/1000 kcal), and iron (7.0 mg/1000 kcal vs. 5.3 mg/1000 kcal) was higher in the T3 group than in the T1 group (all *P* < 0.0001). In contrast, children in the T3 group exhibited the lowest saturated fatty acid intake (12.1 g/1000 kcal vs. 13.5 g/1000 kcal, *P* = 0.085).

**Table 3:** Energy and nutrient intake by tertiles of Taiwanese Children Health Eating Index (TCHEI) among preschool children (*n* = 614).

	Taiwanese Children Health Eating Index, median (interquartile ranges)				
	Total	Tertile 1	Tertile 2	Tertile 3	*P* value^a^
Total energy (kcal)	1324 (668)	1079 (694.7)	1349 (661)	1504 (629)	<0.0001
Protein (% of total energy)	15.5 (4.85)	14.1 (5.5)	15.6 (5.0)	15.9 (3.6)	<0.0001
Protein intake (g/kg.BW)	2.78 (1.81)	2.2 (1.6)	2.7 (1.8)	3.5 (1.7)	<0.0001
Fat (% of total energy)^b^	31.2 (11.0)	31.9 (12.4)	30.8 (10.4)	31.2 (10.4)	0.910
Saturated fatty acid (g/1000 kcal)	12.7 (5.51)	13.5 (6.73)	12.5 (5.20)	12.1 (4.85)	0.085
Polyunsaturated fatty acid (g/1000 kcal)	8.69 (4.86)	8.09 (5.00)	8.84 (4.11)	9.23 (5.80)	0.003
Carbohydrate (% of total energy)	54.2 (12.5)	54.3 (14.9)	54.1 (12.1)	54.1 (11.9)	0.359
Dietary fiber (g)	5.74 (4.1)	4.3 (3.3)	5.4 (3.5)	7.2 (3.7)	<0.0001
Nutrients density (/1000 kcal)					
Vitamin A (RE)(μg)	370 (433)	255 (331)	372 (416)	481 (422)	<0.0001
Vitamin E (TE)(mg)	4.36 (3.37)	3.8 (3.4)	4.4 (3.0)	4.8 (3.5)	0.0004
Vitamin C (mg)	51.1 (70.0)	30.8 (69.4)	44.6 (46.7)	76.4 (70.3)	<0.0001
Vitamin B-1 (mg)	0.68 (0.45)	0.6 (0.5)	0.7 (0.4)	0.7 (0.5)	0.0007
Vitamin B-2 (mg)	0.78 (0.77)	0.7 (0.7)	0.8 (0.8)	0.9 (0.8)	0.0112
Vitamin B-6 (mg)	0.80 (1.98)	0.7 (0.4)	0.8 (0.3)	0.9 (1.9)	<0.0001
Potassium (mg)	1132 (488)	978 (415)	1106 (384)	1280 (494)	<0.0001
Calcium (mg)	289 (290)	224 (251)	289 (276)	380 (277)	<0.0001
Phosphate (mg)	615 (216)	560 (230)	629 (208)	647 (194)	<0.0001
Magnesium (mg)	124 (46.3)	110 (44.2)	123 (34.3)	139 (48.1)	<0.0001
Iron (mg)	6.41 (3.78)	5.3 (3.6)	6.5 (3.5)	7.0 (3.8)	<0.0001
Zinc (mg)	5.05 (1.92)	4.4 (1.8)	5.1 (1.9)	5.6 (1.8)	<0.0001

All data were presented as median (interquartile ranges).

^a^Except for the percentage of dietary fat intake, all data were subjected to Kruskal–Wallis test to evaluate the differences between the TCHEI groups.

^b^Analysis of variance was used to evaluate the difference between fat intake and the TCHEI groups.

Table 4 illustrates the association between the TCHEI score of tertile and medical service utilization in the total population and different age groups (2–3 years and 4–6 years). After adjusting for the covariates (Model 2) in the total population, the T2 group exhibited a significant 18% reduction (95% confidence interval (CI): 0.78–0.87) in overall visits and a 17% reduction (95% CI: 0.78–0.87) in outpatient visits compared to the T1 group. A similar trend was observed in emergency medical service utilization, but it was not statistically significant. For the 2–3-year age group, both T2 and T3 groups exhibited substantial reduction in overall (25% and 16%), outpatient (24% and 15%), and emergency (52% and 58%) visits compared to the T1 group. These findings suggest higher TCHEI score is associated with lower medical service utilization in 2–3-year-old age group. In the 4–6-year-old age group, the T2 group exhibited a 15% reduction (95% CI: 0.80–0.91) in overall and outpatient visits compared to the T1 group, while there was no significant reduction in emergency medical service utilization.

**Table 4:** The association between the tertile of Taiwanese Children Health Eating Index score and medical service utilization among preschoolers.

	Taiwanese Children Health Eating Index								
	Exp (*β*) (95% confidence interval)								
	2–6 yrs (*n* = 614)			2–3 yrs (*n* = 168)			4–6 yrs (*n* = 446)		
	T1	T2	T3	T1	T2	T3	T1	T2	T3
*All disease medical service use (times/year)*									
Total									
Crude model	ref.	0.81 (0.76, 0.85)	0.91 (0.86, 0.95)	ref.	0.77 (0.71, 0.84)	0.79 (0.72, 0.86)	ref.	0.83 (0.77, 0.88)	0.98 (0.92, 1.04)
Model 1	ref.	0.82 (0.78, 0.87)	0.96 (0.91, 1.01)	ref.	0.75 (0.69, 0.83)	0.84 (0.77, 0.93)	ref.	0.86 (0.80, 0.92)	1.03 (0.97, 1.10)
Model 2	ref.	0.82 (0.78, 0.87)	0.96 (0.91, 1.01)	ref.	0.75 (0.69, 0.83)	0.84 (0.77, 0.92)	ref.	0.85 (0.80, 0.91)	1.02 (0.96, 1.09)
Outpatient									
Crude model	ref.	0.81 (0.77, 0.85)	0.91 (0.87, 0.96)	ref.	0.78 (0.71, 0.85)	0.80 (0.73, 0.87)	ref.	0.82 (0.77, 0.88)	0.98 (0.92, 1.04)
Model 1	ref.	0.83 (0.78, 0.87)	0.96 (0.92, 1.01)	ref.	0.76 (0.70, 0.84)	0.86 (0.78, 0.94)	ref.	0.86 (0.80, 0.92)	1.03 (0.96, 1.10)
Model 2	ref.	0.83 (0.78, 0.87)	0.96 (0.91, 1.01)	ref.	0.76 (0.69, 0.84)	0.85 (0.78, 0.94)	ref.	0.85 (0.80, 0.91)	1.02 (0.96, 1.09)
Emergency									
Crude model	ref.	0.68 (0.47, 1.00)	0.73 (0.50, 1.05)	ref.	0.49 (0.27, 0.91)	0.47 (0.26, 0.87)	ref.	0.86 (0.52, 1.40)	0.96 (0.60, 1.54)
Model 1	ref.	0.69 (0.47, 1.01)	0.72 (0.50, 1.05)	ref.	0.48 (0.25, 0.90)	0.42 (0.22, 0.81)	ref.	0.87 (0.52, 1.43)	1.01 (0.62, 1.64)
Model 2	ref.	0.69 (0.47, 1.01)	0.73 (0.50, 1.06)	ref.	0.48 (0.25, 0.90)	0.42 (0.21, 0.81)	ref.	0.87 (0.53, 1.45)	1.04 (0.64, 1.69)
*Respiratory disease medical service use (times/year)*									
Total									
Crude model	ref.	0.82 (0.77, 0.87)	0.91 (0.86, 0.97)	ref.	0.76 (0.68, 0.84)	0.75 (0.67, 0.82)	ref.	0.87 (0.80, 0.94)	1.03 (0.95, 1.11)
Model 1	ref.	0.83 (0.78, 0.89)	0.96 (0.90, 1.02)	ref.	0.73 (0.65, 0.81)	0.79 (0.71, 0.88)	ref.	0.90 (0.82, 0.97)	1.08 (0.99, 1.17)
Model 2	ref.	0.83 (0.78, 0.88)	0.95 (0.90, 1.02)	ref.	0.73 (0.65, 0.81)	0.79 (0.71, 0.88)	ref.	0.89 (0.82, 0.97)	1.07 (0.99, 1.16)
Outpatient									
Crude model	ref.	0.82 (0.77, 0.88)	0.92 (0.86, 0.98)	ref.	0.77 (0.69, 0.85)	0.75 (0.68, 0.83)	ref.	0.87 (0.80, 0.94)	1.03 (0.95, 1.11)
Model 1	ref.	0.84 (0.79, 0.90)	0.96 (0.90, 1.02)	ref.	0.74 (0.66, 0.82)	0.81 (0.72, 0.90)	ref.	0.90 (0.82, 0.97)	1.08 (0.99, 1.17)
Model 2	ref.	0.84 (0.79, 0.89)	0.96 (0.90, 1.02)	ref.	0.74 (0.66, 0.82)	0.80 (0.72, 0.89)	ref.	0.89 (0.82, 0.97)	1.07 (0.98, 1.16)
Emergency									
Crude model	ref.	0.50 (0.26, 0.95)	0.64 (0.36, 1.16)	ref.	0.26 (0.09, 0.73)	0.28 (0.11, 0.75)	ref.	0.91 (0.37, 2.21)	1.26 (0.56, 2.85)
Model 1	ref.	0.49 (0.26, 0.96)	0.63 (0.34, 1.16)	ref.	0.27 (0.09, 0.79)	0.28 (0.10, 0.81)	ref.	0.85 (0.34, 2.14)	1.26 (0.55, 2.90)
Model 2	ref.	0.49 (0.26, 0.96)	0.63 (0.34, 1.16)	ref.	0.27 (0.09, 0.79)	0.28 (0.10, 0.82)	ref.	0.86 (0.34, 2.14)	1.28 (0.56, 2.96)

*T* tertile. Model 1 was adjusted for age, gender, region, mother’s education level and household financial status by multivariable generalized linear model. Model 2 was further adjusted for total energy intake.

The analysis of respiratory disease medical service utilization in the total population revealed that after adjustment for the confounding factors, the T2 group exhibited significant reductions in overall (17%), outpatient (16%), and emergency (51%) visits for respiratory disease compared to the T1 group. For the 2–3-year-old age group, both the T2 and T3 groups exhibited reductions in overall (27% and 21%), outpatient (26% and 20%), and emergency (73% and 72%) visits for respiratory disease compared to the T1 group. In contrast, for the 4–6-year-old age group, the T2 group exhibited an 11% reduction in overall and outpatient medical visits for respiratory disease compared to the T1 group; with no significant reduction observed in emergency medical service utilization for respiratory disease.

Table 5 shows the association between TCHEI score and medical expenditure. In the T2 group, after adjustment for confounding factors, a lower overall medical expenditure rate was exhibited by the total population (exp β: 0.68, 95% CI: 0.57–0.80), 2–3-year-old age group (exp β: 0.72, 95% CI: 0.53–0.96), and 4–6-year-old age group (exp β: 0.68, 95% CI: 0.56–0.84) compared with the T1 group. Regarding the outpatient visit medical expenditure, the participants aged 4–6 years and belonging to the T2 group exhibited 33% (95% CI: 0.54–0.82) lower outpatient visit medical expenditures, but a non-significant association was found in 2–3 years. However, the analysis of respiratory disease medical expenditures revealed that the participants aged 2–3 years and belonging to the T2 group exhibited 28% (95% CI: 0.53–0.97) lower medical expenditures. Furthermore, in the T2 and T3 groups, participants aged 2–3 years exhibited an 87% (95% CI: 0.04–0.37) and 80% (95% CI: 0.06–0.63) reduction in emergency visit medical expenditure.

**Table 5:** The association between the tertile of Taiwanese Children Health Eating Index score and medical expenditure among preschoolers.

	Taiwanese Children Health Eating Index								
	Exp (*β*) (95% confidence interval)								
	2–6 yrs (*n* = 614)			2–3 yrs (*n* = 168)			4–6 yrs (*n* = 446)		
	T1	T2	T3	T1	T2	T3	T1	T2	T3
*All disease medical expenditure (NT$/year)*									
Total									
Crude model	ref.	0.68 (0.57, 0.82)	0.89 (0.75, 1.06)	ref.	0.74 (0.55, 1.01)	0.73 (0.54, 0.99)	ref.	0.65 (0.52, 0.80)	0.98 (0.79, 1.21)
Model 1	ref.	0.68 (0.57, 0.80)	0.98 (0.82, 1.16)	ref.	0.72 (0.53, 0.96)	0.85 (0.63, 1.16)	ref.	0.69 (0.56, 0.85)	1.08 (0.88, 1.32)
Model 2	ref.	0.68 (0.57, 0.80)	0.97 (0.82, 1.15)	ref.	0.72 (0.53, 0.96)	0.85 (0.62, 1.15)	ref.	0.68 (0.56, 0.84)	1.06 (0.87, 1.31)
Outpatient									
Crude model	ref.	0.68 (0.57, 0.82)	0.90 (0.75, 1.07)	ref.	0.77 (0.56, 1.05)	0.75 (0.55, 1.03)	ref.	0.63 (0.51, 0.79)	0.97 (0.78, 1.21)
Model 1	ref.	0.67 (0.57, 0.80)	0.98 (0.82, 1.17)	ref.	0.74 (0.55, 1.01)	0.89 (0.65, 1.22)	ref.	0.67 (0.55, 0.83)	1.06 (0.86, 1.31)
Model 2	ref.	0.67 (0.56, 0.80)	0.97 (0.81, 1.15)	ref.	0.75 (0.55, 1.01)	0.88 (0.64, 1.21)	ref.	0.67 (0.54, 0.82)	1.04 (0.85, 1.28)
Emergency									
Crude model	ref.	0.73 (0.49, 1.09)	0.78 (0.53, 1.15)	ref.	0.49 (0.23, 1.03)	0.52 (0.25, 1.08)	ref.	1.01 (0.64, 1.61)	1.07 (0.68, 1.70)
Model 1	ref.	0.82 (0.54, 1.24)	0.84 (0.55, 1.28)	ref.	0.41 (0.17, 0.98)	0.44 (0.19, 1.02)	ref.	1.12 (0.68, 1.85)	1.16 (0.71, 1.91)
Model 2	ref.	0.82 (0.54, 1.25)	0.86 (0.57, 1.31)	ref.	0.41 (0.17, 0.98)	0.44 (0.19, 1.03)	ref.	1.15 (0.70, 1.89)	1.23 (0.74, 2.03)
*Respiratory disease medical expenditure (NT$/year)*									
Total									
Crude model	ref.	0.80 (0.67, 0.96)	0.89 (0.75, 1.06)	ref.	0.77 (0.57, 1.04)	0.71 (0.53, 0.95)	ref.	0.83 (0.68, 1.02)	1.03 (0.84, 1.26)
Model 1	ref.	0.84 (0.71, 0.99)	0.98 (0.83, 1.16)	ref.	0.72 (0.53, 0.97)	0.78 (0.57, 1.05)	ref.	0.88 (0.72, 1.08)	1.08 (0.88, 1.31)
Model 2	ref.	0.82 (0.69, 0.98)	0.90 (0.76, 1.07)	ref.	0.72 (0.53, 0.97)	0.78 (0.57, 1.05)	ref.	0.88 (0.72, 1.07)	1.07 (0.88, 1.31)
Outpatient									
Crude model	ref.	0.82 (0.69, 0.98)	0.90 (0.76, 1.07)	ref.	0.82 (0.60, 1.10)	0.74 (0.55, 1.00)	ref.	0.83 (0.67, 1.02)	1.02 (0.83, 1.25)
Model 1	ref.	0.86 (0.73, 1.01)	0.99 (0.83, 1.16)	ref.	0.77 (0.57, 1.05)	0.82 (0.61, 1.11)	ref.	0.88 (0.72, 1.08)	1.05 (0.86, 1.29)
Model 2	ref.	0.86 (0.72, 1.01)	0.98 (0.83, 1.16)	ref.	0.77 (0.57, 1.05)	0.82 (0.61, 1.12)	ref.	0.88 (0.72, 1.07)	1.05 (0.86, 1.28)
Emergency									
Crude model	ref.	0.43 (0.28, 0.65)	0.66 (0.44, 1.01)	ref.	0.22 (0.10, 0.48)	0.30 (0.14, 0.65)	ref.	0.91 (0.56, 1.47)	1.49 (0.92, 2.39)
Model 1	ref.	0.61 (0.38, 0.96)	0.96 (0.60, 1.55)	ref.	0.16 (0.06, 0.46)	0.21 (0.07, 0.65)	ref.	0.95 (0.57, 1.60)	1.91 (1.10, 3.31)
Model 2	ref.	0.61 (0.39, 0.96)	0.96 (0.60, 1.55)	ref.	0.13 (0.04, 0.37)	0.20 (0.06, 0.63)	ref.	0.95 (0.56, 1.59)	1.94 (1.12, 3.37)

*T* tertile. Model 1 was adjusted for age, gender, region, mother’s education level and household financial status by multivariable generalized linear model. Model 2 was further adjusted for total energy intake.

We excluded the medical service usage and expenditure regarding accidental injuries and inquired the association between TCHEI score and all medical service utilization. The results remain consistent (Supplementary Table 4). Participants with congenital conditions were excluded and most of finding were similar, however, the medical expenditures in the 2–3-year-old age group were not significant (Supplementary Table 5).

## Discussion

Our study demonstrated that Taiwanese preschoolers, especially toddlers, adhering to the dietary guidelines exhibited higher nutrient intake, lower medical service utilization, and medical expenditure. Hence, adherence to food-based dietary guidelines could contribute to reduce medical service utilization in Taiwanese preschoolers.

Children may incur higher medical service or expenditure due to congenital conditions. Furthermore, all medical utilization includes visits due to accidental injuries which may not be directly related to dietary quality. To account for these factors, a sensitivity analysis was conducted to examine the relationship between dietary quality and all medical service and expenditure. After excluding the participants with congenital conditions, the inversely association between TCHEI and all disease medial expenditure in 2–3-year-old age group became non-significant. For the medical service use, the analysis results revealed consistent findings with our previous analyses (Supplementary Table 5).

The preschoolers with higher dietary intake quality had more educated parents and superior family economic status. In Taiwan, mothers play a crucial role in preparing meals for their family members and 95% reported that mother is primary care of participant (data not shown). Mothers with higher education levels may possess higher nutrition knowledge and abilities to ensure a healthy dietary pattern in their family environment [20, 21]. In addition, family economic status is positively associated with household food accessibility, availability, and affordability. Several studies have demonstrated that children from low-income families exhibit a higher risk of food insecurity and poor dietary diversity [22, 23]. In developed countries, low-income families may force their children to choose low-nutrient but high-energy dense foods, such as ultraprocessed foods [24–26], leading to an increase in the risk of obesity [24]. Obese individuals exhibit a 36% increase in total annual medical costs compared with those with a healthy body weight [27]. Therefore, a poor diet may result in a high risk of chronic diseases and greater medical care utilization in the future [28]. In Taiwan, children under 5 years of age and who are economically disadvantaged exhibit higher outpatient medical service utilization and hospitalization days [29] but lower dental service utilization [30]. In our study, we revealed that children from households with economic statuses categorized as enough, just enough, not enough or very difficult had overweight or obesity rates of 8.45%, 16.4%, and 15.5%, respectively. Furthermore, children classified as overweight or obese exhibited higher median healthcare utilization and medical expenditures (data not shown). However, it was evident that the T2 (16.0%) exhibited a highest prevalence of overweight/obesity, and followed by T1 (14.5%) among the TCHEI groups, but did not reach statistical significance (*P* = 0.768). With only 50 individuals classified as overweight or obese in this limited population, whether overweight or obesity leads to increased medical expenditures still requires further research for confirmation.

Among the preschoolers, TCHEI score was positively associated with total energy and the intake of protein, dietary fiber, polyunsaturated fatty acids, and most vitamins and minerals. The finding is consistent with that of a prospective study in Finland that evaluated dietary quality by using the Finnish Children Healthy Eating Index [31]. The intake of dietary fiber, polyunsaturated fatty acids, and vitamin C reduces inflammation in the human body. Furthermore, sufficient energy obtained from food and protein intake is the key to physiological growth and also is reflected in height. Our study demonstrated that participants with higher TCHEI scores exhibited a greater HAZ score than did those with lower TCHEI scores. HAZ score is an indicator of the long-term nutritional status of children under 5 years of age [32]. A Bangladesh-based study revealed that children aged 24–59 months with higher dietary diversity exhibited a 31% lower risk of stunting than those with lower dietary diversity [33]. Furthermore, children aged 6–35 months with higher Infant and Child Feeding Index scores exhibited a 23% lower risk of stunting and 24% lower undernutrition in China [34]. In the Maldives, the risk of stunting and undernutrition was inversely associated with HAZ scores for children aged 6–59 months [35]. Therefore, practicing a healthy dietary pattern to obtain sufficient energy and nutrients can reduce the risk of abnormal growth and development, which in turn may reduce medical service utilization in early life.

This study mainly revealed that preschoolers, especially children aged 2–3 years, with optimal diet quality exhibited reduced medical usage and expenditure for outpatient and emergency of overall and respiratory disease. The immune system is not sound in children below the age of 4 years, leading to a higher chance of using medical care services compared with children aged 4–6 years. A study identified Taiwan’s NHI medical service as high users used, defined as more than 80% of the insurers’ medical expenses in the year, and low users used less than 80% of the medical expenses. The author reported that the proportion of high users aged 0–4 years accounted for 19.3% of total users [36]. A healthy diet could lower healthcare financial burden among this age group which can as evidence for our nutritional promotion policy. However, among those age 4–6, the U-shape of association between TCHEI score and medical utilization was found, with T2 having the lowest medical utilization and expenditure. Healthcare service utilization is influenced by various factors, including family economic status, life behavior, and residential environment. Despite adjusting for maternal education level and household financial status in the model, the influence of these factors cannot be entirely ruled out.

A Canadian cross-sectional study explored the association between a healthy diet and medical expenditures and the number of medical visits in grade 5 children. The results revealed that the third tertile group in the Diet Quality Index or Healthy Eating Index exhibited lower (4–7%) medical expenditures and number of medical visits (6%) than the first tertile group did [11]. Although the Canadian study exhibited a trend, the results were non-significant; this may be because of different sample, healthcare accessibility or parents’ medical seeking behaviors.

Our study found that children aged 2–3 with the lowest TCHEI group had the highest mean annual outpatient expenditure (NT$12159 ≒ US$392) and emergency expenditure (NT$1189 ≒ US$38) in all disease. These children also had the highest mean annual outpatient expenditure (NT$ 6121 ≒ US$197) and emergency expenditure (NT$793 ≒ US$25) in respiratory disease (Supplementary Table 3). We did not include indirect cost such as caregivers’ productivity loss. This could as an example that poor diet could increase medical cost. The economic benefit of saving medical costs through potentially modifiable risk factors such as poor diet could be a promoting healthy diet strategy.

## Strengths and limitations

The study was conducted by a prospective design and the coverage rate of Taiwan’s NHI program is 99.9%. Therefore, these population-based data provide comprehensive information on medical service utilization for each visit. Furthermore, the data collection was conducted by systematic measurements of national nutrition monitoring system with robust sample size.

This study has the following limitations. First, the dietary pattern obtained by the 24-h diet recall method may not represent the usual dietary pattern. However, the content of children’s diet is relatively monotonous [37], without much variation in the short term. Therefore, even single-day data can still represent participants’ daily intake. Second, the index was developed in accordance with the TFBDG, and the results of our study do not incorporate weighting. As a result, these findings are applicable solely to our sample and may not be generalized to other populations. Third, physical activity, living environment (air pollution, sanitization, medical accessibility), and psychological stress are crucial indicators of health; however, we did not collect these information, leading to the presence of residual confounders.

## Conclusion

We developed the TCHEI according to the TFBDG for preschoolers. Preschoolers adhering to the dietary guidelines exhibited higher nutrient intake, a healthy body weight, and lower medical service utilization.

### Supplementary information

Supplementary tables

## Data Availability

The dataset of this study is available from the Health and Welfare Data Science Center, Ministry of Health and Welfare (Taiwan), but restrictions apply to the availability of these data, which were used under approval for the current study, and so are not publicly available.
