# Correction: Adherence to dietary guidelines associated with lower medical service utilization in preschoolers: a longitudinal study

**DOI:** 10.1038/s41387-024-00279-1

**Published:** 2024-04-15

**Authors:** Yi-Chieh Chen, Yuan-Ting C. Lo, Hsin-Yun Wu, Yi-Chen Huang

**Affiliations:** 1 Department of Nutrition, China Medical University, No. 100, Sec. 1, Jingmao Rd., Beitun District, Taichung, 406040 Taiwan; 2 Department of Nutrition, Chang Bing Show Chwan Memorial Hospital, No. 6, Lugong Rd., Lukang Township, Changhua County, 50544 Taiwan; 3 Department of Public Health, National Defense Medical Center, No. 161, Sec. 6, Minquan E. Rd., Niehu District, Taipei, 11490 Taiwan

**Keywords:** Paediatrics, Epidemiology

Correction to: *Nutrition and Diabetes* 10.1038/s41387-024-00270-w, published online 22 March 2024

The author identified an error regarding the Institutional Review Board (IRB) approval number. In the study participants and ethical approval sections, the IRB approval number was incorrectly listed as ‘CRRE-110-002’. The correct number should be ‘CRREC-108-157’.

The original article has been corrected.

