# Supplementary material for: Adherence to dietary guidelines associated with lower medical service utilization in preschoolers: a longitudinal study
Source: Nutr Diabetes. 2024 Mar 22;14:11. doi: 10.1038/s41387-024-00270-w (PMC10959943; doi:10.1038/s41387-024-00270-w)
Supplement: Supplementary file 1 — Supplementary tables [file 41387_2024_270_MOESM1_ESM.docx]

Supplementary Table 1. Baseline characteristics of total and subpopulation dataset

|  | Total population^a^  (n=1295) | Subpopulation^b^  (n=614) | *P* value |
| --- | --- | --- | --- |
| Age (years) |  |  | 0.78 |
| 2 | 193 (14.9) | 93 (15.2) |  |
| 3 | 177 (13.7) | 75 (12.2) |  |
| 4 | 324 (25.0) | 145 (23.6) |  |
| 5 | 308 (23.8) | 150 (24.4) |  |
| 6 | 293 (22.6) | 151 (24.6) |  |
| Gender, boy | 657 (50.7) | 327 (53.3) | 0.30 |
| Region |  |  | 0.16 |
| North | 429 (33.1) | 204 (33.2) |  |
| Middle | 201 (15.5) | 100 (16.3) |  |
| South | 400 (30.9) | 163 (26.6) |  |
| East or island | 265 (20.5) | 147 (23.9) |  |
| Mother’s education level |  |  | 0.80 |
| Under junior high school | 134 (10.5) | 68 (11.2) |  |
| Senior high school | 436 (34.2) | 200 (32.8) |  |
| Above college | 706 (55.3) | 342 (56.1) |  |
| Father’s education level |  |  | 0.55 |
| Under junior high school | 151 (11.9) | 75 (12.6) |  |
| Senior high school | 462 (34.5) | 202 (33.9) |  |
| Above college | 653 (51.6) | 319 (53.5) |  |
| Household financial status |  |  | 0.66 |
| Enough | 229 (17.9) | 121 (19.9) |  |
| Just enough | 714 (55.9) | 336 (55.3) |  |
| Not enough | 262 (20.5) | 122 (20.1) |  |
| Very difficulty | 73 (5.7) | 29 (4.8) |  |

^a^ Total population refer to the entire NAHSIT

^b^ Subpopulation includes only those consent to be linked for medical utilization dataset.

Supplementary Table 2. Scores of each component by tertiles of Taiwanese Children Health Eating Index among preschool children

|  | Taiwanese Children Health Eating Index (TCHEI) | | | | |
| --- | --- | --- | --- | --- | --- |
|  | Total | T1 | T2 | T3 | *P* value |
| Overall dietary quality score | 50.610.6 | 38.75.6 | 50.72.5^*^ | 61.85.4^*^ | <0.0001 |
| Dietary adequacy score (75) | 34.610.8 | 23.47.0 | 34.94.6^*^ | 45.26.2^*^ | <0.0001 |
| Grains (15) | 8.42.8 | 6.83.0 | 8.92.4^*^ | 9.52.2^*^ | <0.0001 |
| Grains (10) | 7.72.5 | 6.42.7 | 8.12.3^*^ | 8.61.9^*^ | <0.0001 |
| Unrefined grains (5) | 0.71.2 | 0.40.9 | 0.71.2^*^ | 0.91.3^*^ | <0.0001 |
| Soy/fish/eggs/meat (15) | 9.73.6 | 7.54.0 | 10.22.9^*^ | 11.52.6^*^ | <0.0001 |
| Soy/fish/eggs/meat (10) | 8.32.9 | 6.73.7 | 8.72.4^*^ | 9.31.5^*^ | <0.0001 |
| Soy products (5) | 1.52.0 | 0.81.6 | 1.51.9^*^ | 2.22.1^*^ | <0.0001 |
| Dairy (10) | 3.53.5 | 2.73.3 | 3.43.3 | 4.53.7^*^ | <0.0001 |
| Vegetables (15) | 6.24.4 | 2.82.9 | 6.33.9^*^ | 9.43.6^*^ | <0.0001 |
| Vegetables (10) | 3.83.1 | 1.61.7 | 3.92.9^*^ | 5.92.9^*^ | <0.0001 |
| Dark vegetables (5) | 2.42.3 | 1.22.0 | 2.52.3^*^ | 3.52.0^*^ | <0.0001 |
| Fruits (10) | 3.13.6 | 1.32.4 | 2.63.1^*^ | 5.33.8^*^ | <0.0001 |
| Oils and nuts (10) | 3.73.0 | 2.32.4 | 3.52.8^*^ | 5.13.2^*^ | <0.0001 |
| Eating behavior score (25) | 15.94.0 | 15.34.5 | 15.83.9 | 16.63.4^*^ | 0.005 |
| Eating breakfast (5) | 4.61.4 | 4.02.0 | 4.71.1^*^ | 4.90.7^*^ | <0.0001 |
| Frequency of SSB (5) | 0.31.1 | 0.31.1 | 0.10.9 | 0.41.4 | 0.08 |
| Frequency of fried food (5) | 3.92.1 | 3.42.3 | 3.92.1 | 4.31.8^*^ | 0.0002 |
| Frequency of snack/ biscuits (5) | 4.90.8 | 4.81.1 | 4.90.9 | 5.00.5 | 0.06 |
| Frequency of seasoning (5) | 2.32.5 | 2.82.5 | 2.22.5 | 2.02.5^*^ | 0.005 |

Analysis of variance was used to evaluate the difference between each component and the TCHEI groups. Bonferroni correction was used for post-hoc comparison. The reference group was T1. ^*^ *P* < 0.017

Supplementary Table 3. Mean annual medical care utilization and expenditure by tertiles of Taiwanese Children Health Eating Index among preschooler

|  | Taiwanese Children Health Eating Index | | | | | | |
| --- | --- | --- | --- | --- | --- | --- | --- |
|  | All disease | | |  | Respiratory disease | | |
|  | Tertile 1 | Tertile 2 | Tertile 3 |  | Tertile 1 | Tertile 2 | Tertile 3 |
| Medical service use (times/year) | |  |  |  |  |  |  |
| 2-6 yrs (n=614) |  |  |  |  |  |  |  |
| Total | 16.0 (13.0) | 13.2 (11.0) | 15.3 (12.4) |  | 10.4 (7.79) | 8.7 (6.53) | 10.0 (7.62) |
| Outpatient | 15.6 (12.5) | 12.9 (10.9) | 15.1 (12.3) |  | 10.2 (7.76) | 8.6 (6.47) | 9.8 (7.52) |
| Emergency | 0.32 (0.18) | 0.22 (0) | 0.23 (0) |  | 0.15 (0.14) | 0.07 (0.07) | 0.09 (0.08) |
| 2-3 yrs (n=168) |  |  |  |  |  |  |  |
| Total | 21.2 (17.4) | 16.0 (15.5) | 17.9 (15.9) |  | 15.8 (12.0) | 11.5 (10.6) | 12.5 (11.3) |
| Outpatient | 20.4 (16.3) | 15.6 (14.8) | 17.5 (15.4) |  | 15.2 (11.4) | 11.2 (10.6) | 12.3 (11.3) |
| Emergency | 0.71 (0.37) | 0.34 (0) | 0.30 (0) |  | 0.43 (0) | 0.12 (0) | 0.12 (0) |
| 4-6 yrs (n=446) |  |  |  |  |  |  |  |
| Total | 13.5 (11.1) | 11.6 (9.52) | 13.9 (11.4) |  | 8.0 (6.44) | 7.2 (5.78) | 8.7 (6.77) |
| Outpatient | 13.3 (10.7) | 11.4 (9.32) | 13.7 (11.0) |  | 8.0 (6.32) | 7.1 (5.67) | 8.6 (6.77) |
| Emergency | 0.19 (0) | 0.16 (0) | 0.19 (0) |  | 0.05 (0) | 0.04 (0) | 0.07 (0) |
| Medical expenditure (NT$^a^/year) | |  |  |  |  |  |  |
| 2-6 yrs (n=614) |  |  |  |  |  |  |  |
| Total | 9763.4 (5645.3) | 6251.0 (4521.5) | 9244.6 (5477.9) |  | 4328.2 (2998.9) | 3541.5 (2630.4) | 4047.3 (3023.9) |
| Outpatient | 9138.9 (5114.5) | 5813.3 (4072.7) | 8750.8 (5133.3) |  | 4013.0 (2958.7) | 3381.2 (2550.6) | 3820.7 (2941.2) |
| Emergency | 543.1 (135.9) | 394.6 (2.26) | 425.9 (2.00) |  | 247.2 (1.49) | 106.0 (1.42) | 162.9 (1.56) |
| 2-3 yrs (n=168) |  |  |  |  |  |  |  |
| Total | 13421.3 (9161.2) | 10037.7 (6796.5) | 10686.1 (7084.3) |  | 7040.3 (5032.5) | 5176.6 (4488.2) | 5364.2 (4684.4) |
| Outpatient | 12159.9 (6918.6) | 9415.4 (6419.4) | 10040.7 (6612.5) |  | 6121.7 (4919.2) | 4813.6 (4416.1) | 4963.2 (4647.6) |
| Emergency | 1189.4 (691.4) | 568.0 (2.31) | 587.5 (1.96) |  | 793.9 (2.21) | 177.9 (1.48) | 243.9 (1.58) |
| 4-6 yrs (n=446) |  |  |  |  |  |  |  |
| Total | 7880.1 (4596.7) | 4861.0 (3939.4) | 8503.2 (5177.1) |  | 3558.9 (2462.9) | 3117.2 (2226.3) | 3789.5 (2621.9) |
| Outpatient | 7506.3 (4176.5) | 4505.3 (3550.2) | 8081.2 (4848.9) |  | 3046.7 (2315.1) | 2633.8 (2122.9) | 3214.4 (2508.6) |
| Emergency | 303.0 (634.0) | 304.1 (688.7) | 343.7 (817.2) |  | 73.7 (1.46) | 61.0 (1.41) | 107.0 (1.56) |

Data was presented as mean (median). The mean was adjusted for age, gender, region, mother’s education level and household financial status by multivariable generalized linear model.

^a^ NT$, with an exchange rate about NT$ 31 to US$ 1 in 2024.

Supplementary Table 4. The association between the tertile of Taiwanese Children Health Eating Index score and medical service utilization (excluding the related to accidental injuries) among preschooler

|  | | Taiwanese Children Health Eating Index | | | | | | | | | | | | |
| --- | --- | --- | --- | --- | --- | --- | --- | --- | --- | --- | --- | --- | --- | --- |
|  | | Exp ($\beta$) (95% confidence interval) | | | | | | | | | | | | |
|  | | 2-6 yrs (n=614) | | | | |  | 2-3 yrs (n=168) | | |  | 4-6 yrs (n=446) | | |
|  | | T1 | | T2 | | T3 |  | T1 | T2 | T3 |  | T1 | T2 | T3 |
| **All disease medical service use (times/year)** | | | | | | |  |  |  |  |  |  |  |  |
| *Total* | | | | | |  |  |  |  |  |  |  |  |  |
| Crude model | | ref | | 0.81 (0.76, 0.85) | | 0.91 (0.86, 0.95) |  | ref | 0.77 (0.71, 0.84) | 0.79 (0.72, 0.86) |  | ref | 0.83 (0.77, 0.88) | 0.98 (0.92, 1.04) |
| Model 1 | | ref | | 0.82 (0.78, 0.87) | | 0.96 (0.91, 1.01) |  | ref | 0.75 (0.69, 0.83) | 0.84 (0.77, 0.93) |  | ref | 0.86 (0.80, 0.92) | 1.03 (0.97, 1.10) |
| Model 2 | | ref | | 0.82 (0.78, 0.87) | | 0.96 (0.91, 1.01) |  | ref | 0.75 (0.69, 0.83) | 0.84 (0.77, 0.92) |  | ref | 0.85 (0.80, 0.91) | 1.02 (0.96, 1.09) |
| *Outpatient* | |  | |  | |  |  |  |  |  |  |  |  |  |
| Crude model | | ref | | 0.81 (0.77, 0.85) | | 0.91 (0.87, 0.96) |  | ref | 0.78 (0.71, 0.85) | 0.80 (0.73, 0.87) |  | ref | 0.83 (0.77, 0.88) | 0.98 (0.92, 1.04) |
| Model 1 | | ref | | 0.83 (0.78, 0.87) | | 0.96 (0.92, 1.02) |  | ref | 0.76 (0.70, 0.84) | 0.86 (0.78, 0.94) |  | ref | 0.86 (0.80, 0.92) | 1.03 (0.97, 1.10) |
| Model 2 | | ref | | 0.82 (0.78, 0.87) | | 0.96 (0.91, 1.01) |  | ref | 0.76 (0.69, 0.84) | 0.85 (0.78, 0.94) |  | ref | 0.85 (0.79, 0.91) | 1.02 (0.96, 1.09) |
| *Emergency* | |  | |  | |  |  |  |  |  |  |  |  |  |
| Crude model | | ref | | 0.67 (0.46, 0.98) | | 0.73 (0.50, 1.05) |  | ref | 0.48 (0.26, 0.88) | 0.47 (0.26, 0.87) |  | ref | 0.85 (0.52, 1.40) | 0.96 (0.60, 1.55) |
| Model 1 | | ref | | 0.67 (0.46, 0.99) | | 0.72 (0.50, 1.06) |  | ref | 0.46 (0.24, 0.87) | 0.42 (0.22, 0.80) |  | ref | 0.86 (0.52, 1.43) | 1.02 (0.63, 1.66) |
| Model 2 | | ref | | 0.68 (0.46, 1.00) | | 0.73 (0.50, 1.06) |  | ref | 0.46 (0.24, 0.87) | 0.42 (0.22, 0.81) |  | ref | 0.87 (0.52, 1.45) | 1.05 (0.64, 1.71) |
| **All disease medical expenditure (NT$/year)** | | | | | | |  |  |  |  |  |  |  |  |
| *Total* |  | |  | |  | |  |  |  |  |  |  |  |  |
| Crude model | ref | | 0.68 (0.57, 0.82) | | 0.89 (0.75, 1.06) | |  | ref | 0.74 (0.55, 1.00) | 0.73 (0.54, 0.99) |  | ref | 0.65 (0.52, 0.80) | 0.98 (0.79, 1.21) |
| Model 1 | ref | | 0.68 (0.57, 0.80) | | 0.98 (0.82, 1.16) | |  | ref | 0.71 (0.53, 0.96) | 0.85 (0.63, 1.16) |  | ref | 0.69 (0.56, 0.85) | 1.08 (0.88, 1.32) |
| Model 2 | ref | | 0.68 (0.57, 0.80) | | 0.97 (0.82, 1.15) | |  | ref | 0.71 (0.53, 0.96) | 0.85 (0.62, 1.15) |  | ref | 0.69 (0.56, 0.85) | 1.06 (0.87, 1.31) |
| *Outpatient* |  | |  | |  | |  |  |  |  |  |  |  |  |
| Crude model | ref | | 0.68 (0.57, 0.82) | | 0.90 (0.75, 1.07) | |  | ref | 0.77 (0.57, 1.05) | 0.75 (0.55, 1.03) |  | ref | 0.63 (0.51, 0.79) | 0.97 (0.78, 1.21) |
| Model 1 | ref | | 0.68 (0.57, 0.80) | | 0.98 (0.82, 1.17) | |  | ref | 0.74 (0.55, 1.01) | 0.89 (0.65, 1.22) |  | ref | 0.67 (0.55, 0.83) | 1.06 (0.86, 1.31) |
| Model 2 | ref | | 0.67 (0.56, 0.80) | | 0.97 (0.81, 1.16) | |  | ref | 0.74 (0.55, 1.01) | 0.88 (0.64, 1.21) |  | ref | 0.67 (0.54, 0.82) | 1.04 (0.85, 1.29) |
| *Emergency* |  | |  | |  | |  |  |  |  |  |  |  |  |
| Crude model | ref | | 0.71 (0.48, 1.06) | | 0.78 (0.52, 1.15) | |  | ref | 0.45 (0.21, 0.95) | 0.52 (0.25, 1.09) |  | ref | 1.02 (0.64, 1.61) | 1.07 (0.68, 1.70) |
| Model 1 | ref | | 0.81 (0.53, 1.23) | | 0.84 (0.55, 1.28) | |  | ref | 0.38 (0.16, 0.92) | 0.43 (0.18, 1.01) |  | ref | 1.14 (0.69, 1.88) | 1.18 (0.72, 1.94) |
| Model 2 | ref | | 0.81 (0.54, 1.24) | | 0.87 (0.57, 1.32) | |  | ref | 0.38 (0.16, 0.91) | 0.43 (0.18, 1.02) |  | ref | 1.17 (0.71, 1.92) | 1.24 (0.75, 2.05) |

Model 1 was adjusted for age, gender, region, mother’s education level and household financial status by multivariable generalized linear model. Model 2 was further adjusted for total energy intake.

Supplementary Table 5. The association between the tertile of Taiwanese Children Health Eating Index score and medical service use and medical expenditure among preschooler without congenital disease

|  | Taiwanese Children Health Eating Index | | | | | | | | | | | |
| --- | --- | --- | --- | --- | --- | --- | --- | --- | --- | --- | --- | --- |
|  | Exp ($\beta$) (95% confidence interval) | | | | | | | | | | | |
|  | 2-6 yrs (n=570) | | | |  | 2-3 yrs (n=151) | | |  | 4-6 yrs (n=419) | | |
|  | T1 | | T2 | T3 |  | T1 | T2 | T3 |  | T1 | T2 | T3 |
| **All disease medical service use (times/year)** | | | | |  |  |  |  |  |  |  |  |
| *Total* | | | |  |  |  |  |  |  |  |  |  |
| Crude model | ref | | 0.82 (0.77, 0.86) | 0.93 (0.88, 0.98) |  | ref | 0.84 (0.76, 0.92) | 0.85 (0.78, 0.94) |  | ref | 0.81 (0.76, 0.86) | 0.96 (0.90, 1.03) |
| Model 1 | ref | | 0.83 (0.79, 0.88) | 0.98 (0.93, 1.04) |  | ref | 0.82 (0.74, 0.90) | 0.90 (0.81, 0.99) |  | ref | 0.83 (0.78, 0.89) | 1.02 (0.95, 1.09) |
| Model 2 | ref | | 0.83 (0.79, 0.88) | 0.98 (0.93, 1.03) |  | ref | 0.81 (074, 0.90) | 0.89 (0.80, 0.99) |  | ref | 0.83 (0.77, 0.89) | 1.01 (0.94, 1.08) |
| *Outpatient* |  | |  |  |  |  |  |  |  |  |  |  |
| Crude model | ref | | 0.82 (0.78, 0.86) | 0.93 (0.88, 0.98) |  | ref | 0.85 (077, 0.93) | 0.86 (0.79, 0.95) |  | ref | 0.80 (0.75, 0.86) | 0.96 (0.90, 1.02) |
| Model 1 | ref | | 0.83 (0.79, 0.88) | 0.98 (0.93, 1.04) |  | ref | 0.83 (0.75, 0.92) | 0.91 (0.82, 1.01) |  | ref | 0.83 (0.78, 0.89) | 1.02 (0.95, 1.09) |
| Model 2 | ref | | 0.83 (0.79, 0.88) | 0.98 (0.93, 1.04) |  | ref | 0.82 (0.77, 0.91) | 0.91 (0.82, 1.01) |  | ref | 0.83 (0.77, 0.89) | 1.01 (0.94, 1.08) |
| *Emergency* |  | |  |  |  |  |  |  |  |  |  |  |
| Crude model | ref | | 0.71 (0.48, 1.05) | 0.77 (0.52, 1.14) |  | ref | 0.51 (0.27, 0.96) | 0.49 (0.26, 0.94) |  | ref | 0.88 (0.53, 1.46) | 1.01 (0.62, 1.66) |
| Model 1 | ref | | 0.72 (0.48, 1.08) | 0.79 (0.53, 1.18) |  | ref | 0.52 (0.26, 1.00) | 0.46 (0.23, 0.94) |  | ref | 0.90 (0.54, 1.51) | 1.12 (0.67, 1.86) |
| Model 2 | ref | | 0.72 (0.48, 1.08) | 0.80 (0.53, 1.19) |  | ref | 0.52 (0.26, 1.00) | 0.47 (0.23, 0.95) |  | ref | 0.90 (0.54, 1.52) | 1.14 (0.68, 1.90) |
| **All disease medical expenditure (NT$/year)** | | | | |  |  |  |  |  |  |  |  |
| *Total* |  |  | |  |  |  |  |  |  |  |  |  |
| Crude model | ref | 0.69 (0.57, 0.83) | | 0.91 (0.76, 1.10) |  | ref | 0.80 (0.58, 1.11) | 0.77 (0.55, 1.06) |  | ref | 0.64 (0.51, 0.80) | 0.98 (0.79, 1.23) |
| Model 1 | ref | 0.68 (0.57, 0.81) | | 0.99 (0.83, 1.19) |  | ref | 0.79 (0.58, 1.08) | 0.91 (0.66, 1.27) |  | ref | 0.68 (0.55, 0.85) | 1.09 (0.88, 1.35) |
| Model 2 | ref | 0.68 (0.57, 0.81) | | 0.99 (0.82, 1.18) |  | ref | 0.79 (0.58, 1.08) | 0.91 (065, 1.27) |  | ref | 0.68 (0.55, 0.84) | 1.08 (0.87, 1.34) |
| *Outpatient* |  |  | |  |  |  |  |  |  |  |  |  |
| Crude model | ref | 0.68 (0.57, 0.83) | | 0.92 (0.76, 1.11) |  | ref | 0.82 (0.59, 1.14) | 0.79 (0.56, 1.10) |  | ref | 0.62 (0.50, 0.78) | 0.98 (0.78, 1.23) |
| Model 1 | ref | 0.67 (0.56, 0.80) | | 0.99 (0.82, 1.19) |  | ref | 0.81 (0.58, 1.12) | 0.94 (0.67, 1.32) |  | ref | 0.66 (0.53, 0.82) | 1.07 (0.86, 1.33) |
| Model 2 | ref | 0.67 (0.56, 0.80) | | 0.98 (0.81, 1.18) |  | ref | 0.81 (0.58, 1.12) | 0.93 (0.93, 1.16) |  | ref | 0.65 (0.53, 0.81) | 1.05 (0.84, 1.31) |
| *Emergency* |  |  | |  |  |  |  |  |  |  |  |  |
| Crude model | ref | 0.83 (0.55, 1.25) | | 0.86 (0.57, 1.29) |  | ref | 0.57 (0.26, 1.26) | 0.54 (0.25, 1.20) |  | ref | 1.09 (0.68, 1.75) | 1.17 (0.73, 1.89) |
| Model 1 | ref | 0.93 (0.60, 1.44) | | 0.95 (0.61, 1.48) |  | ref | 0.52 (0.20, 1.38) | 0.50 (0.20, 1.27) |  | ref | 1.23 (0.73, 2.06) | 1.31 (0.78, 2.20) |
| Model 2 | ref | 0.93 (0.60, 1.43) | | 0.95 (0.61, 1.48) |  | ref | 0.54 (0.20, 1.42) | 0.52 (0.20, 1.31) |  | ref | 1.23 (0.74, 2.06) | 1.33 (0.79, 2.25) |

Model 1 was adjusted for age, gender, region, mother’s education level and household financial status by multivariable generalized linear model. Model 2 was further adjusted for total energy intake.
